# Nerve Tire in the Schools

**Published:** 1899-11

**Authors:** 


### SELECTIONS.

### Nerve Tire in the Schools.

Half a million of New York's population are now busily engaged over book and slate. It is no unimportant part of any community's inhabitants, this youthful element for whom each fall the vast machinery of education is set in motion.

Physicians and public-health officials have a duty in the matter which teachers, school commissioners and parents may at times overlook. All interested in the wellfare of the growing generation should pause to consider the question of possible over-training. A physical or mental wreck is a most depressing sight at any period of life, but a break-down at the very threshold, when foresight on the part of others might have prevented it, is sad indeed.

No doubt one of the chief causes of nerve fatigue in school children is lack of sufficient fresh air, and especially of bodily exercise in it. In the desire to crowd facts and figures into the receptive brain, the instructors, and behind them the commissioners or committeemen, lose sight of that most important fact, that the building of a beautiful character and a physically strong body is the most essential part of the whole education. Where overcrowding prevails in the home life and unhygienic surroundings predispose to conditions of anemia, the school should all the more endeavor to counteract the evil influences by supplying, so far as possible, the elements of health which are lacking.

Time to breathe a little outside air, time to stretch the muscles, and, above all, time to eat the mid-day meal slowly and to digest it partially before returning to the desk, should be provided for the students.

Dr. Scheidt, who has given much attention to school hygiene, delivered a long address last year before the Associated Sanitary Authorities of Pennsylvania, of which some of the conclusions reached were these :

"1. I am convinced that the esthesiometer is the proper instrument for the measurement of fatigue.

"2. My tentative experiments with American boys prove a superior normal strength of their mental vigor under favorable conditions of the atmosphere.

"3. Unfavorable conditions of the atmosphere show an unusually large percentage of abnormal fatigue of the nervous system.

"4. I attribute this condition to the utter lack of systematic open-air exercises.

"5. I advocate such exercises for children as a prime necessity, because the foundation of a healthy and useful life are laid between the seventh and fifteenth years, but never afterward.

"6. Training of all the functions of the physical organism should precede instruction, because it will develop the necessary mental strength.

"7. Children should be classified according to their individual characteristics, and stress should be laid upon the development of their weakest organs, in such a way, however, as to leave plenty of room for individualization.

"8. Such exercise should take place every afternoon, and should be continued until the limit of normal fatigue is reached.

"9. The exercises should be grouped into muscular, cutaneous and respiratory.

"10. All these exercises should be essentially exercises of the nervous system, to lead to a proper development of character."

Speaking of the part the physician should take in this matter, Dr. Scheidt says: "The chief factor in every school board should be the well-trained physician." When this suggestion is adopted we may look forward to a lessening of that nerve fatigue, irritability, and final collapse which is so often brought about by poor digestion, absence of open-air playground, and the punishment by "keeping in." Of all barbarous practices, that of allowing twenty minutes for a cold lunch, and then detaining a young child after hours in a vitiated atmosphere because indigestion and beginning dyspepsia have produced irritability and inattention is the most fruitful source of nervous weariness which sooner or later leads to the physical wreck.—*N. Y. Med. Record.*

